# Hemophagocytic Lymphohistiocytosis: an Unusual Complication of *Orientia tsutsugamushi* Disease (Scrub Typhus)

**DOI:** 10.4084/MJHID.2015.008

**Published:** 2015-01-01

**Authors:** Aneesh Basheer, Somanath Padhi, Vinoth Boopathy, Saumyaranjan Mallick, Shashikala Nair, Renu G’Boy Varghese, Reba Kanungo

**Affiliations:** 1 Department of General Medicine, Pondicherry Institute of Medical Sciences, Puducherry, India.; 2 Department of Pathology, Pondicherry Institute of Medical Sciences, Puducherry, India.; 3 Department of Gastroenterology, Pondicherry Institute of Medical Sciences, Puducherry, India.; 4 Department of Pathology, All India Institute of Medical Sciences, New Delhi, India.; 5 Department of Microbiology, Pondicherry Institute of Medical Sciences, Puducherry, India.

## Abstract

**Background:**

Hemophagocytic lymphohistiocytosis (HLH) is an uncommon, potentially fatal, hyperinflammatory syndrome that may rarely complicate the clinical course of *Orientia tsutsugamushi* disease (scrub typhus).

**Methods:**

Here we describe the clinicopathological features, laboratory parameters, management, and outcome of three adult patients (1 female, 2 males) with scrub typhus associated HLH from a tertiary center. A brief and concise review of international literature on the topic was also added.

**Results:**

All three patients satisfied the HLH-2004 diagnostic criteria; one had multi-organ dysfunction with very high ferritin level (>30,000 ng/ml), and all had a dramatic recovery following doxycyclin therapy. Literature review from January 1990 to March 2014 revealed that scrub typhus associated HLH were reported in 21 patients, mostly from the scrub endemic countries of the world. These included 11 females and 10 males with a mean age of 35 years (range; 8 months to 81 years). Fifteen of 17 patients (where data were available) had a favorable outcome following early serological diagnosis and initiation of definitive antibiotic therapy with (N=6) or without (N=9) immunosuppressive/immunomodulator therapy. Mutation analysis for primary HLH was performed in one patient only, and HLH-2004 protocol was used in two patients.

**Conclusion:**

We suggest that HLH should be considered in severe cases of scrub typhus especially if associated with cytopenia (s), liver dysfunction, and coagulation abnormalities. Further studies are required to understand whether an immunosuppressive and/or immunomodulator therapy could be beneficial in those patients who remain unresponsive to definitive antibiotic therapy.

## Introduction

Hemophagocytic lymphohistiocytosis (HLH) is a syndrome, often fatal, of exacerbated but ineffective inflammatory response, characterized by excessive macrophage and T-cell activation as well as impairment of the ability of natural killer (NK) cell and cytotoxic T lymphocytes to kill the target cells. This results in uncontrolled histiocytic phagocytosis of mature blood elements and their precursors throughout the reticuloendothelial organs; and associated cytokine-mediated multiorgan dysfunction.^1^,^2^ Primary or familial HLH appears to have a genetic basis, whereas secondary or acquired HLH may be associated with infections (commonly Epstein Barr Virus, bacteria, Rickettsia, etc), hematological malignancies (mostly T/NK cell leukemias/lymphomas), rheumatological/autoimmune disorders (so-called macrophage activation syndrome), etc.^3^–^5^ The diagnosis is established by fulfilling one of the following HLH-2004 criteria: i) positive family history or molecular diagnosis consistent with HLH (mutations of PRF, SAP, or MUNC13-4 genes), ii) any five out of the following eight criteria: prolonged fever, unexplained progressive cytopenias involving at least 2 cell lines (hemoglobin ≤ 90 g/L, platelet count ≤ 100 × 10^9^/L, absolute neutrophil count < 1 × 10^9^/L), splenomegaly, hyperferritinemia (≥ 500ng/mL), fasting hypertriglyceridemia (≥ 265 mg/dL) or hypofibrinogenemia (≤ 1.5 g/L), histiocytic hemophagocytosis in bone marrow, liver, spleen, or lymph nodes without evidence of malignancy, low or absent NK cell cytotoxicity, and elevated soluble CD25 levels (≥ 2400 IU/mL of interleukin-2Rα chain).^6^

In this manuscript, we describe three adult patients with scrub typhus associated HLH from a tertiary care center in South India. We also present a brief concise review of international literature regarding scrub typhus associated HLH in relation to the clinicopathological characteristics, immunopathology, and therapeutic outcome.

## Material and Methods

### Present case descriptions

The clinicopathological features, laboratory data, and therapeutic outcome of all three patients (1 female, aged 19 years; and 2 males, aged 64, 45 years) are presented in Table 1. All the three required admission into intensive care unit. They had prolonged fever (that ranged from 10 days to 2 months duration) and generalized signs, and symptoms suggestive of systemic inflammatory response syndrome (SIRS), which were presumptively managed as sepsis. All three developed tachypnea, bilateral coarse crepitations and hypoxemia. Clinical, laboratory, and radiological features were consistent with acute respiratory distress syndrome (ARDS), which required ventilator support. In addition, two of the patients (cases 1 & 2) also developed acute kidney injury; all three had jaundice with elevated liver transaminases with deranged coagulation parameters in the form of prolonged prothrombin time (PT) and/or activated partial thromboplastin time (APTT); and one patient (case 1) progressed to disseminated intravascular coagulation (DIC) [increased fibrin degradation product, D-dimer + (qualitative)], possibly secondary to severe sepsis. Physical examination revealed conjunctival pallor and hepatosplenomegaly in all; whereas lymphadenopathy (cervical and axillary) and an *eschar* (over left groin) were noted in one patient (case 3).

Microbiological and serological work-up was negative for HIV, hepatitis B and C viruses, dengue, malaria, leptospira, brucella, and Epstein-Barr virus. Microbial cultures of blood, urine, sputum, bronchoalveolar lavage fluid, and bone marrow aspirate were sterile. A diagnosis of scrub typhus was confirmed by presence of IgM antibody against *Orientia tsutsugamushi (O. tsutsugamushi)* by ELISA. All underwent single bone marrow procedure due to persistent bi/pancytopenia, fever, and organomegaly; that revealed histiocytic hemophagocytosis (marked in case 1, mild in cases 2 & 3). The assessment of histiocytic hemophagocytosis on bone marrow aspirate smears was done as per the method devised by Ho et al (low/mild; 1–5 hemophagocytic cells per entire slide, moderate; 6–10 hemophagocytic cell per entire slide; and > 10 hemophagocytic cell per entire slide).^7^ Furthermore, all three had hyperferritinemia (>1000 ng/mL) [a “sky high” level of >30,000 ng/mL in case 1] and elevated lactate dehydrogenase (>500 IU/L); and two (cases 1 & 2) had hypertriglyceridemia (>265 mg/dL), hypoalbuminemia (<2.5 g/dL), and hyponatremia (≤ 125 meq/L). All patients fulfilled five of six HLH-2004 diagnostic criteria, as NK cell activity and soluble CD25 levels were not tested due to lack of facility, and *none of the patients were subjected to genetic testing for the diagnosis of primary HLH*.

All 3 patients were empirically treated with broad-spectrum intravenous antibiotics and supportive measures. Following confirmation of scrub typhus by IgM ELISA, oral doxycyclin (100 mg twice daily) was started. Furthermore, case 1 was started on intravenous corticosteroids and vasopressor drugs. Over a period of one week, the patients responded positively to the treatment, with abatement of fever, resolution of acute kidney injury, and hepatic dysfunction with correction of coagulopathy. Considering the very high ferritin level and clinical scenario in case 1, hematologist’s opinion was sought for commencing HLH-2004 protocol. After resolution of multiorgan dysfunction (MOD), etoposide was added to the ongoing regimen and was later withdrawn after full recovery of clinical and hematological parameters. At present this patient is under regular follow-up without any medications. The other two patients did not receive any specific immunosuppressive therapy for HLH and improved following treatment with oral doxycyclin.

## Review of International Literature

A systematic search of HLH that complicated the course of scrub typhus over last 24 years (January 1990 to March 2014) was done by the search engine of PubMed, PubMed Central, Medline, and Directory of Open Access Journal databases. The following terminologies were used in the data search; hemophagocytic lymphohistiocytosis/hemophagocytosis/erythrophagocytosis/ macrophage activation syndrome and scrub typhus/*Orientia tsutsugamushi*/tsutsugamushi disease/Rickettsial disease; and references of all articles were cross-checked for relevant articles. Review showed 18 papers describing 21 cases of scrub typhus associated HLH around the world.^5^,^8^–^25^ Cascio et al, in 2011, reported a case of HLH (negative for perforin 1, MUNC 13–4, SYNTAXIN 11 mutations) in a 5 year old Italian male child secondary to *Rickettsia conorii* (Mediterranean Spotted Fever, MSF); and reviewed another 16 cases of rickettsia associated HLH till that year [7 due to scrub typhus, 5 due to MSF, and 4 due to human monocytic ehrlichiosis (HME)].^5^ In 4 of 21 cases of scrub typhus (2 from Taiwan, two from Japan), complete articles were not fully accessible for study.^19^,^22^,^25^ Clinical and laboratory data available have been put in a tabular format (Table 2).

## Result and Discussion

Scrub typhus, also known as tsutsugamushi disease, is a chigger-borne zoonosis which is of great public health importance in tropical Asia and the islands of the Western Pacific Ocean. This infection or its etiologic agent (*O. tsutsugamushi*) has been documented within an endemic triangle with apices in the Primorje region of the Russian Far East, northern Australia, and Afghanistan.^26^ Scrub typhus is an acute febrile illness which results from the bite of infected larval form of mite, called chigger, in endemic areas. Following an incubation period of 7 to 10 days, the nonspecific prodrome of pyrexia, skin rash, myalgia, gastrointestinal disturbances, and lymphadenopathy starts. Although not consistently seen, the most pathognomonic sign of scrub typhus is an *eschar* that develops at the site of mite bite. During human infection, *O. tsutsugamushi,* being a rickettsial organism, selectively targets the vascular endothelial cells of the small to medium-sized blood vessels. However, it can also invade underlying tissues such as smooth muscle cells, perivascular macrophages, and monocytes. Consequently, widespread vasculitis/perivasculitis is the hallmark pathophysiologic mechanism implicated in multiorgan dysfunction syndrome (MODS) in patients with severe infections.^5^,^26^

The main pathophysiologic characteristic of HLH is exacerbated but deregulated Th_1_ cell-mediated immune response against an intracellular pathogen, macrophage hyperactivity, widespread hemophagocytosis, and hypercytokinemia leading to multi-organ dysfunction. This results due to impaired or suppressed function of cytotoxic T cells and NK cells to effectively clear the antigenic stimulus and thus turn off the inflammatory response.^1^ The severity of rickettsial diseases varies with the causative organism and the host. Some rickettsial species such as *Rickettsia rickettsii*, *Rickettsia prowazekii*, and *O. tsutsugamushi* often cause more severe disease. Host factors like old age, alcoholism, diabetes mellitus, liver and respiratory diseases, and glucose-6-phosphate dehydrogenase deficiency have been associated with more severe disease.^5^ Experimental studies have shown that human cells are capable of controlling rickettsial infections intracellularly, by one or combination of three mechanisms involving nitric oxide synthesis, hydrogen peroxide production, and tryptophan degradation. These mechanisms involve a complex interaction of CD4^+^ and CD8^+^ T lymphocytes, macrophages, NK cells, B lymphocytes, antibodies, and cytokines. Inflammatory responses of humans appear to coincide with the disease severity in scrub typhus, and the cytotoxic T-cell mediated macrophage over activity may induce hemophagocytosis in susceptible individuals.^27^ This hypothesis has been further substantiated by the observation of increased serum levels of IFN- γ, M-CSF and TNF-α in patient with scrub typhus in several studies.^28^

A concise review on clinicopathological characteristics of all reported cases of scrub typhus associated HLH till March 2014 is presented in Table 2. There were 21 reported cases with HLH, complicating the course of scrub typhus (7 from India, 5 from China, 4 from Japan, 4 from South Korea, and 1 from Sri Lanka).^5^,^8^–^25^ These included 11 females and 10 males, with a mean age of 35 years (range; 8 months to 81 years); and none of these cases had obvious underlying co-morbidities. All patients, 18/18, in whom data were available, presented with acute febrile illness (mean duration; 10 days, range: 4 to 24 days): similarly all cases reported (14/14) had hepatosplenomegaly with or without regional lymphadenopathy. A characteristic eschar was signaled in 9/18 (50%) patients and seven of 18 (39%) had ARDS, 3 (16.7%) had neurological manifestations, 2 (11%) had acute renal failure, 2 (11%) had DIC with MODS, 2 (11%) had at onset acute hepatic failure. One had co-existent leptospirosis,^8^ and one had myocarditis, interstitial pneumonia, and reactivation of EBV infection.^13^ Eleven of 18 patients (61%), in whom data were available, had bicytopenia; 4/18 (22%) had pancytopenia, and 6/13 (37.5%) had coagulation abnormalities. Histiocytic hemophagocytosis was reported in bone marrow of all 16 examined patients (100%). Hemophagocytosis was of mild to moderate intensity in 14 (87.5%) patients whereas appeared marked in only two patients. This was reflected by modest elevation of serum ferritin level (N=13/21) (mean; 3320 ng/ml, range; 1415–15,000 ng/ml). *Molecular analysis for primary HLH such as mutation of perforin and SYNTAXIN gene was performed in one patient (8 month male child) only*.^11^ A recent study on pediatric HLH patients (N=38; 20 primary HLH, 18 secondary HLH) reported that hyperbilirubinemia with cholestasis was highly suggestive of primary HLH; whereas high C-reactive protein levels was more in favor of a secondary HLH. The other parameters such as ferritin, triglyceride, PT, APTT were not statistically different between the two groups.^29^ All the 3 patients in our series satisfied the HLH-2004 criteria and had hyperbilirubinemia (marked in case 1) with biochemical evidence of cholestasis (direct bilirubin fraction greater than indirect), two had raised transaminases and all three had coagulation abnormality in the form of prolonged PT, APTT, or both (Table 1). One patient (case 1) had “sky high” ferritin levels (>30,000 ng/ml), high procalcitonin levels suggestive of sepsis, and evidence of marked degree of histiocytic hemophagocytosis in bone marrow. During the clinical course, she progressed to DIC with hypofibrinogenemia leading to MODS.

Serological diagnosis of scrub typhus is usually possible after 5 to 10 days following onset of symptoms. Conventionally, Weil-Felix test based on heterophile antigen of *Proteus Vulgaris* (OX-2, and OX-19) and *Proteus mirabilis* (OX-K) has been used widely. An agglutinating titer of >320 with OX-K is suggestive of scrub typhus.^30^ However, several Indian studies have shown a lower cut-off titer at ≥ 80 to indicate a possible infection with *O. tsutsugamushi,* thus making the test even more non-specific.^31^,^32^ Poor sensitivity and specificity of Weil-Felix is well established. Recently, IgM ELISA and IgM Capture ELISA techniques have shown better predictive value as diagnostic tests in scrub typhus. PCR-based tests have overcome the drawbacks of serological tests, in early detection using specific primers;^33^ and multiplex PCR has further improved diagnostic criteria in endemic areas.^34^ In all the reported cases of scrub typhus associated HLH, the diagnosis of rickettsial disease was serologically confirmed. The following serological methods were used for the diagnosis: Weil-Felix (N=5), IgM ELISA (N=6), IgM antibodies by indirect haemagglutination (IHA) (N=1), antibody titres by immunofluorescence (N=3), IgM antibody by immunochromatography (N=1), and IgM anti *O. tsutsugamushi* Gilliam antibody (N=1).

Scrub typhus responds more readily to antibiotics than other rickettsial diseases, with most patients becoming afebrile within 24 to 36 hours after beginning antibiotic therapy. A recent study by Japanese group had shown that age was an independent risk factor for mortality; furthermore patients with ≥ 2 days delay in treatment with tetracyclines had a significantly higher risk of complications compared to those without delay.^35^ In another study from South Korea, factors such as old age, presence of co-morbidities, and high serum osteopontin (>100ng/ml) were important risk factors of disease severity; though delay in treatment and strain type (Boryong, Taguchi, or Kanda/Kawasaki) did not contribute to disease severity.^36^ Therapeutic and follow-up data were available In 17 patients with scrub typhus associated HLH, ten of them received oral antibiotics in the form of doxycyclin and/or clarithromycin/minocyclin; six patients received immunotherapy in addition to oral antibiotic therapy (two of them used HLH-2004), 12,13 and one patient refused any definitive therapy.^15^ Barring two patients (one with residual neurological impairment,^12^ and other with fatal outcome,^15^ all 15 had a dramatic recovery from their illness without any residual impairment.

Though the HLH protocol has been well established in the management of primary HLH, its utility in the setting of secondary HLH has been controversial.^2^ High ferritin level has been reported to be a diagnostic and prognostic marker in patients with HLH, and a rapid rate of fall of ferritin levels following therapy initiation associated with decreased mortality.^37^ However, Park et al. in their cohort of 23 patients with secondary HLH found that high fibrinogen at the time of diagnosis, not the rate of decline in ferritin, was associated with prolonged survival. Furthermore, in patients with severe disease and/or associated sepsis or multiple organ failure at the time of diagnosis, it may be difficult to use cytotoxic agents such as etoposide. In such circumstances, immunosuppression with corticosteroids and/or cyclosporine remains the foundation of early management as it can control systemic inflammation.^38^ In view of the limited number of cases of HLH at our institute; etoposide has not been widely used; hence limiting our experience. A very high ferritin (>30, 000 ng/ml), in conjunction with other criteria, was highly characteristic of HLH in one of our patients (case 1). During the course of her illness, she developed MODS secondary to sepsis and DIC (prolonged PT, aPTT, and increased fibrin degradation product) (Table 1). She was managed initially with vasopressor drugs, high-dose corticosteroids, and intravenous broad-spectrum antibiotics. Doxycyclin was added following the serological diagnosis of scrub typhus. Once her clinical conditions improved, etoposide was added in consultation with hematologist; however, it was later withdrawn after restoration of normal hematological parameters.

## Summary

HLH though rare, should be considered in severe cases of scrub typhus especially if associated with cytopenias, liver dysfunction, and coagulation abnormalities. *Furthermore, wherever possible, a diagnosis of primary HLH should always be excluded in these cases by appropriate mutation analysis studies.* As observed in our three patients and supported by available literature, early diagnosis and initiation of definitive antibiotic therapy may completely reverse the clinical course of the disease**.** Further studies are needed to understand whether an immunosuppressive and/or immunomodulator therapy such as treatment with corticosteroids, etoposide or cyclosporine could be beneficial in those cases which do not respond promptly to tetracycline therapy.

## Figures and Tables

**Table 1:** Clinicopathological characteristics and outcome data of 3 adult patients with scrub typhus associated hemophagocytic lymphohistiocytosis (HLH) diagnosed as per HLH-2004 criteria.^6^

Patientcharacteristics	Case 1	Case 2	Case 3
Age (years)/gender	19, female	64, male	45, male
Presumptive diagnosis	?Sepsis	Sepsis, MODS	Sepsis
HLH-2004 criteria			
Fever	+ (3 weeks)	+ (10 days)	+ (15 days), eschar+
Splenomegaly ± hepatomegaly	+	+	+, lymph nodes
Cytopenia (≥ 2 cell lines)	+	+	+
• Hemoglobin ≤ 90g/L	+ (66 g/L)	+ (70)	+ (69)
• Plateletcount ≤ 100 × 10^9^/L	+(80 × 10^9^/L)	+ (76)	+ (10)
• Absolute neutrophilcount (<1000/L)	+ (675)	− (3700)	+ (250)
Fasting serum triglyceride (> 265 mg/dL)	+ (760)	+ (399)	not tested
Hypofibrinogenemia (<150 mg/L)	not tested at diagnosis	not tested	not tested
Hyperferritinemia (> 500 ng/ml)	+ (>30,000)	+ (1354)	(>2000)
Hemophagocytosis in bone marrow [7]	marked	mild-moderate	mild-moderate
Natural Killer cell activity	not done	not done	not done
Soluble CD25	notdone	not done	not done
Moleculartesting	notdone	notdone	not done
Liver transaminases (≥ 200IU/L)	+	+ (240/90)	WNL
Hyperbilirubinemia (mg/dL) (TB/DB)	+ (22.5/17.5)	+ (9/7.9)	+ (4.9/3.9)
Prolonged PT ± aPTT (seconds)	+ (55/90)	+ (−/50.4)	+ (45/76)
Lactatedehydrogenase (IU/L)	3000	760	530
Hypoalbuminemia (< 3.5 g/dL)	+ (2.5)	+ (2.2)	−
Hyponatremia (< 135 meq/L)	+ (126)	+ (122)	−
D-dimer (semi-quantitative) and FDP	strongly positive	not done	+
Antinuclearantibody	−	not done	notdone
Procalcitonin (ng/dL)	+ (>100)	not done	notdone
Etiology screen	*Orientia tsutsugamushi* (IgM ELISA). Secondary bacterial sepsis	*Orientia tsutsugamushi* (IgM ELISA)	*Orientia tsutsugamushi* (IgM ELISA)
Management	IV antibiotics, doxycyclin, ventilator support, vasopressor, steroids, etoposide	Doxycyclin	Doxycyclin
Outcome	Alive, on follow-up, 12 months	Cured and discharged	Cured and discharged

**Abbreviations:** MODS; multiorgan dysfunction syndrome, +; present, WNL; within normal limits, TB/DB; total bilirubin/direct bilirubin, −; not present, FDP; fibrin degradation product, IV; intravenous.

**Table 2:** Clinicopathological characteristics of hemophagocytic lymphohistiocytosis (HLH) in association with *Orientia tsutsugamushi* disease (Scrub typhus) (March 2014-January 1990) (N=18).^8^–^18^, ^21^–^25^

Age, gender, presentation Eschar/rash	Fever (days)	Spleen	Hb, g/L	Plt, ×10^9^/L	ANC, /cmm	Ferritin, ng/mL	TG, mg/ dL	Fibrinogen, g/L	HP in BM	ALT/AST//LDH	Diagnostic method	Therapy, Outcome (days)	Place of reporting, year, [ref.]
40yr, female, ARDS, seizure	Yes (10)	Yes	77	15	WNL	4662	733	ND	Present	404/300/2140	IgM ELISA (Leptospira), Weil-Felix	Dx + Mp; cured (14)	India, 2014 [8]
20yr, male, ARDS, ALF, eschar+	Yes (14)	Yes	97	200	WNL	ND	ND	ND	BM notdone	150/265/601	Weil-Felix	Dx, cured (15)	India, 2013 [9, 10]
8 month male child ARDS, CNS, DIC, eschar+	Yes (10)	Yes	71	78	High	7970	WNL	53 mg/dl, FDP-36mcg/ml	BM notdone	340/598/10,600	IgMantibody(IHA test)	Cl + Dex+ Ep, afebrile (4), cured (35)	South Korea, 2013 [11]
9yr, female child CNS, eschar & rash +	Yes (7)	Yes	99	77	3000	>7690	WNL	WNL	Moderate	114/200/715	IgM ELISA	HLH-2004 protocol, Dx, neurological impairment	South Korea, 2012 [12]
7yr, male child Myocarditis, interstitial pneumonia, liver dysfunction, Rash +	Yes (4)	Yes	79	17	2240	>1650	377	Low	Present	101/157/1193	IgM, IgG, EBNA (EBV) IgM antibody (ICT)	HLH-2004, clarithromycin, cured (90)	South Korea, 2012 [13]
5yr, female child, ARDS, ARF, Eschar & rash +	Yes (6)	Yes	113	100	1000	4435	335	Low	Mild	114/156/NA	Weil-Felix, IgM ELISA	Dx, cured (7)	India, 2011 [14]
81yr, female, MODS	Yes (NA)	Yes	69	72	7300	1530	NA	Low	Moderate	112/91/2174	IgM ELISA	Supportive, antibiotics, death (35).	South Korea, 2010 [15]
35yr, male, ARDS Eschar +	Yes (10)	Yes	70	13	1500	15000	250	NA	Marked	179/98/NA	IgM ELISA	Dx, cured (10)	India, 2010 [16]
61yr, male, ARF	Yes (20)	Yes	68	75	700	1500	226	NA	Mild	326/132/NA	IgM ELISA	Dx, cured (3)	India, 2010 [16]
23yr, male, non specific	Yes (5)	Yes	120	47	1700	3700	265	NA	Mild	198/155/NA	IgM ELISA	Dx, cured (2)	India, 2010 [16]
22yr, male, FUO, rash	Yes (10)	Yes	85	12	238	>2000	-	NA	Mild	NA	Weil-Felix	Dx, cured (2)	India,2010 [17]
58yr, female, ARDS Eschar+	Yes (24)	Yes	65	56	782	NA	WNL	WNL	Moderate	WNL	IgG ELISA (IF)	Dx, cured (14)	Sri Lanka, 2009 [18]
75yr, female, FUO Eschar&rash +	Yes (NA)	NA	128	14	WNL	183	NA	NA	Moderate	32/44/686	IgM, IgG (IF)	Dx+ Pn, cured (25)	China, 2002 [21]
69yr, female, diahhroea Eschar&rash +	Yes (7)	NA	113	75	Low	282	NA	NA	Mild	17/35/608	IgM, IgG antibody (IF)	Mn, cured (10)	China, 2002 [21]
53yr, female, MODS, DIC Rash +	Yes (NA)	NA	NA	NA	NA	NA	NA	NA	NA	NA		Dx, cured (10)	Japan, 2001 [22]
21yr, male, FUO	Yes (14)	Yes	93	209	Low (3000)	1415	176	NA	Moderate	166/140/1555	Weil-Felix (HA)	Dx, cured (14)	Taiwan, China, 2000 [23]
53yr, female, FUO Eschar +	Yes (7)	Yes	NA	63	Low	NA	NA	NA	Mild	NA/128/760	IgM anti *R tsutsugamushi* Gilliam antibody +	Mn, afebrile (3), cured (90)	Japan, 1994 [24]
47yr, female, ARDS	Yes (NA)	NA	NA	Low	Low	NA	NA	NA	Marked	NA	NA	NA	Japan, 1992 [25]
**Presentseries**													
19yr, female, Sepsis, MODS	Yes (21)	Yes	66	80	675	30000	760	-	Moderate	300/350/3000	IgM ELISA	Steroids, Dx, Ep, alive 12 months	Puducherry, India, 2012
47yr, male, FUO Eschar&rash +	Yes (15)	Yes	69	10	250	>2000	-	low	Mild	−/−/605/530	IgM ELISA	Supportive, Dx	Puducherry, India, 2012
64yr, male, sepsis, MODS	Yes (10)	Yes	70	76		1354	399	-	Moderate	240/90/760	IgM ELISA	Dx, supportive, cured (15)	Puducherry, India, 2013

**Abbreviation:** ARDS; acute respiratory distress syndrome, ALF; acute liver failure, CNS; central nervous system, DIC; disseminated intravascular coagulation, ARF; acute renal failure, MODS; multiorgan dysfunction syndrome, FUO; fever of unknown origin, NA; not available, −; not present or not reported, +; present, Hb; hemoglobin, Plt; platelet count, ANC; absolute neutrophil count, WNL; within normal range, ND; not done/not tested, TG; serum triglyceride, FDP; fibrin degradation product, HP; hemophagocytosis, BM; bone marrow, ALT; alanine aminotransferase, AST; aspartate aminotransferase, LDH; lactate dehydrogenase, ICT; immunochromatography test, IHA; indirect haemagglutination, IF; immunofluorescence, R tsutsugamushi; Rickettsia tsutsugamushi (now called as Orientia tsutsugamushi), Dx; doxycyclin, Mp; methylprednisolone, Cl; clarithromycin, Dex; dexamethasone, Ep; etoposide, Pn; prednisolone, Mn; minocyclin. Note that in only one of 24 cases (including present series), genetic/mutation study was performed for primary HLH (Perforin/syntaxin mutation negative).^11^ Three cases from references^19^, ^20^ where complete information was not available were not presented in the table.
